# Early-Life Stress Caused by Maternal Deprivation Impacts Dendritic Morphology of Adult Male Mouse Neocortical Interneurons

**DOI:** 10.3390/ijms26051909

**Published:** 2025-02-23

**Authors:** Mohammed M. Nakhal, Lidya K. Yassin, Shaikha Al Houqani, Ayishal B. Mydeen, Marwa F. Ibrahim, Safa Shehab, Mohammed Z. Allouh, Mohammad I. K. Hamad

**Affiliations:** Department of Anatomy, College of Medicine and Health Sciences, United Arab Emirates University, Al Ain P.O. Box 15551, United Arab Emirates; 202170013@uaeu.ac.ae (M.M.N.); 700037572@uaeu.ac.ae (L.K.Y.); 202203070@uaeu.ac.ae (S.A.H.); a.mydeen@uaeu.ac.ae (A.B.M.); marwa.i@uaeu.ac.ae (M.F.I.); s.shehab@uaeu.ac.ae (S.S.); m_allouh@uaeu.ac.ae (M.Z.A.)

**Keywords:** maternal deprivation, early-life stress, dendritic growth, interneuron, GAD67, neocortex

## Abstract

A substantial body of research suggests that early-life stress (ELS) is associated with neuropathology in adulthood. Maternal deprivation (MD) is a commonly utilised model in mice for the study of specific neurological diseases. The appropriate growth of dendrites is essential for the optimal functioning of the nervous system. However, the impact of ELS on interneuron dendritic morphology remains unclear. To ascertain whether ELS induces alterations in the morphology of GABAergic inhibitory interneurons in layers II/III of the medial entorhinal cortex (mEC), the somatosensory cortex (SSC), the motor cortex (MC), and the CA1 region of the hippocampus (Hp), 9-day-old male GAD-67-EGFP transgenic mice were subjected to a 24 h MD. At postnatal day 60 (P60), the animals were sacrificed, and their brains were subjected to morphological analyses. The results indicated that MD affected the dendritic morphology of GABAergic interneurons. The mean dendritic length and mean dendritic segments of the examined cortical areas, except for the MC, were significantly decreased, whereas the number of primary dendrites was unaffected. Furthermore, the density of GAD67-EGFP-positive interneurons was decreased in the mEC and Hp, but not in the somatosensory and MC. The induction of ELS through MD in a developmental time window when significant morphological changes occur rendered the developing cells particularly susceptible to stress, resulting in a significant reduction in the number of surviving interneurons at the adult stage.

## 1. Introduction

The early life period is particularly susceptible to environmental influences, including stress, which can have a lasting impact on later life [1]. Early-life stress (ELS) models are designed to replicate stressors such as deprivation, maltreatment, and maternal neglect, which collectively contribute to human adversity [2,3]. These models have been widely utilised over the past two decades, demonstrating significant associations with aberrant brain plasticity, leading to cognitive dysfunction, neurodevelopmental disorders, mood disturbances, and neurodegenerative diseases [4,5]. Maternal deprivation (MD) is a commonly used paradigm for investigating neurobiological alterations associated with stress vulnerability in animals. The MD model involves separating neonatal pups from their mothers for a prolonged period, typically 24 h, at postnatal day 9 (P9). During this separation, the pups are deprived of maternal care and nutrition [6]. This acute stressor results in elevated plasma corticosterone levels, which disrupt postnatal development [6]. Studies have shown that MD can lead to a reduction in body weight, delays in neurological milestones such as negative geotaxis reversal and eye opening, and motor behaviours such as walking and rearing, along with altered responses to amphetamine [7]. These findings suggest that MD can delay neurodevelopment and provide insights into the neurodevelopmental hypothesis of psychiatric disorders, which posits that disruptions in early brain development underlie later psychiatric conditions such as schizophrenia and other psychotic disorders. On a cellular level, MD has been shown to reduce neuronal density, as indicated by a decrease in NeuN-positive neurons and parvalbumin (Parv)-expressing interneurons in the prefrontal cortex (PFC) [8,9]. Additionally, MD reduces the overall number of NeuN-expressing neurons in the amygdala and nucleus accumbens [10]. More recently, MD has been shown to decrease the density and size of calcium-binding protein-expressing interneurons, including Parv-, calbindin-, and calretinin-expressing interneurons, in the amygdala and nucleus accumbens [11]. While these findings suggest that MD can influence interneuron density, it remains unclear whether MD affects interneuron morphology.

Several MD protocols have been proposed, yet evidence suggests that MD at P9–10 is particularly critical in determining adverse outcomes [12]. The age window during which MD is performed is crucial for understanding its effects. Research has demonstrated that MD at P9, compared to MD at P3 or P6, is more effective in modelling early-life stress effects on information processing and cognitive function in adulthood [13]. Furthermore, MD at P9, but not before P4 or after P14 (a period corresponding to stress hyporesponsivity in the hypothalamic–pituitary–adrenal axis), has been shown to impair emotional long-term potentiation (LTP) reinforcement in adolescent animals [14]. The cellular effects of MD are also more pronounced when MD is conducted at P9 compared to P4 [15]. Importantly, MD should not be considered merely as a separation stressor; rather, it constitutes a combination of multiple stressors, including nutritional deprivation. A sharp decline in leptin levels during MD at P9 has been shown to induce sex-specific modifications in hypothalamic trophic factors and cell turnover [16]. Thus, while each stressor contributes to the MD model, their combined effect is the most influential in shaping neurodevelopmental outcomes.

The neocortex relies on a highly interconnected neuronal network composed of two primary neuron types: glutamatergic excitatory neurons (80% of neocortical neurons) and GABAergic inhibitory interneurons (20%) [17]. The activity of interneurons plays a crucial role in modulating neocortical information processing. Anatomically, GABAergic interneurons exhibit diverse morphologies with fewer spines compared to excitatory neurons [18,19,20]. Molecularly, interneurons display substantial variability, and transgenic rodent models expressing fluorescent proteins or recombinase enzymes under interneuron-specific promoters have facilitated their classification based on molecular markers [21]. The three primary non-overlapping markers for neocortical interneurons include Parv, somatostatin, and 5HT3a receptors [22,23]. These three subtypes collectively account for nearly all GABAergic neurons in the somatosensory cortex [24,25]. The impact of stress on interneurons is influenced by multiple factors, including intensity, duration, and controllability, as well as the developmental stage at which stress occurs [26]. For instance, corticosterone exposure for 20 min significantly alters the passive properties of the basolateral amygdala (BLA), increasing excitability while reducing GABA(_A_)-mediated inhibitory postsynaptic currents [27]. Similarly, dexamethasone application in rat hippocampal slices results in heightened synaptic inhibition, disrupting inhibitory network function and rhythmic oscillations [28]. Chronic restraint stress for 10 days induces long-term alterations in cortical interneuron plasticity, involving key molecular players such as the polysialylated form of neural cell adhesion molecule (PSA-NCAM) and perineuronal nets (PNN) [29]. Furthermore, a single 24 h restraint stress event reduces Parv-positive interneuron density in the BLA, highlighting the long-term impact of acute stress on inhibitory circuits [30]. Acute stress has also been shown to impair hippocampus-dependent spatial memory while increasing the density of c-fos-positive interneurons in the CA1 region [31]. Additionally, stress-induced changes in the ventral tegmental area (VTA) GABAergic system may contribute to altered reward-seeking behaviour [32]. Collectively, these findings suggest that acute stress has profound effects on GABAergic interneuron, influencing emotional and cognitive functions. However, the long-term impact of early acute stress on interneuron morphology remains unclear.

Direct human studies on the impact of maternal deprivation on interneurons are lacking, but neuroimaging studies have reported a reduction in GABA levels in the human nucleus accumbens following situational stress [33]. Additionally, early-life stress has been linked to structural brain changes, including reduced grey matter volume in the medial prefrontal cortex, hippocampus, and amygdala [34,35,36]. Given the abundant expression of glucocorticoid receptors in the hippocampus and neocortical areas such as mEC, SSC, and MC [37], we postulated that acute MD, performed at P9 for 24 h, would result in adverse effects on interneuronal growth through extensive glucocorticoid release caused by stress [15,16]. Neuronal dendritic hypertrophy can result in the pathological hyperexcitability of neuronal circuits, which may contribute to the development of disorders such as epilepsy, schizophrenia, bipolar disorder, or autism spectrum disorders [38,39]. The optimal development of interneurons in these cortical regions is of significant biological importance. For example, the dysfunction of interneurons in the mEC has been linked to deficits in spatial navigation, semantic memory, episodic memory, and working memory, which may contribute to the development of a range of neurological disorders [40]. Disruptions in dendritic growth can compromise neuronal circuit formation, leading to pathological hyperexcitability and various neurological disorders. Despite numerous studies examining the effects of MD on brain function, the long-term impact of early stress on interneuron dendritic morphology remains unclear. We hypothesise that MD at P9 disrupts dendritic growth in specific cortical regions, with potential consequences for later cognitive and emotional function.

## 2. Results

### 2.1. MD Reduces the Complexity of the Proximal and Medial Dendrites of Medial Entorhinal Cortical Interneurons

**Figure 1:**
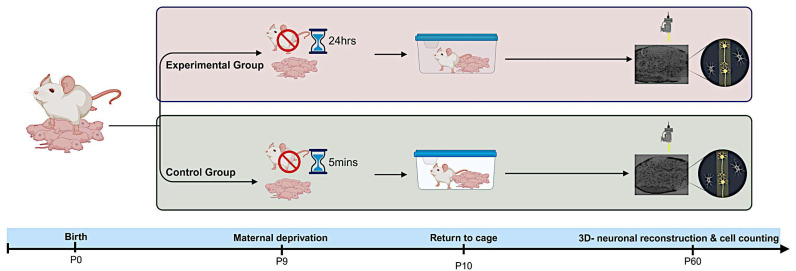
Experimental procedure. The day of delivery was P0. On P9, the litters were subjected to the MD procedure. The dams were removed from the litter, and the pups remained in their home cage at room temperature. A day later, the dams were returned to their cages. As a control experiment, the dams were removed from their home cages for 5 min. The animals were sacrificed at P60, perfused transcardially, immunostained against EGFP, and the dendritic morphology was quantified.

**Figure 2:**
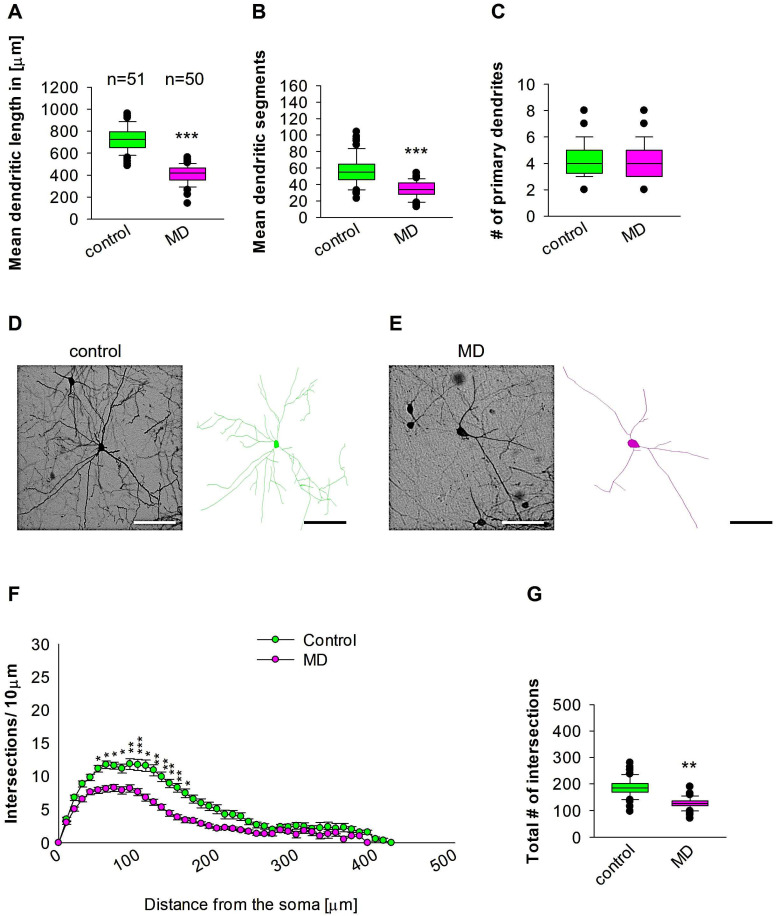
MD effect on the morphology of the interneuron of the mEC. Parasagittal brain slices from the mEC region were stained with EGFP to visualise morphology by light microscopy. GAD67-positive interneurons were reconstructed using Neurolucida. (**A**–**C**,**G**) The median value is represented by the horizontal lines within the box plots, while the variabilities outside the upper and lower quartiles are indicated with whiskers. The middle half of the sample is represented with the box. (**A**) Mean dendritic length. (**B**) Mean dendritic segments. (**C**) Mean of the number of primary dendrites. The number of reconstructed cells is shown in (**A**) for the control and MD groups. The number of cells analysed was obtained from 8 control mice (2–3 slices corresponding only to the mEC area) and 8 MD mice (2–3 slices). Example images at 40× magnification from a control (**D**) and an MD interneuron are shown (**E**). The traces are shown next to the images. Scale bars = 50 μm. (**F**) Sholl analysis of the control and MD groups. The error bars in (**F**) represent the standard error mean. MD-induced decrease in the number of intersections was observed between 60 and 170 μm from the soma using *t*-test post hoc Bonferroni corrections. * *p* < 0.05, ** *p* < 0.01, and *** *p* < 0.001. (**G**) The mean of the total number of dendritic intersections. In the statistical analyses conducted for the experiment in (**F**), the number of dendritic intersections was compared between the control and MD groups at each distance point. For the graphs in (**A**–**C**,**G**), the *p* values were calculated using the Mann–Whitney U test and reported only if they were statistically significant. ** *p* < 0.01; *** *p* < 0.001.

To compare dendritic morphology in the medial entorhinal cortical interneurons between maternally deprived mice and control, we stained the brains with anti-EGFP antibodies to reconstruct 3D dendritic morphology under light microscopy (Figure 1). The staining with anti-EGFP served to convert the fluorescent dye to a stable DAB staining to prevent bleaching over time. The quantitative morphological analyses demonstrate that MD at the early developmental stage of P9 results in a reduction in the dendritic length of GAD67-positive interneurons at the later stage of P60 in comparison to the control group (Figure 2A,D,E). In a similar fashion, the mean dendritic segments of GAD67-positive interneurons from the maternally deprived mice were also decreased in comparison to the interneurons from the control group (Figure 2B,D,E). However, the number of primary dendrites was unaltered between the two groups (Figure 2C–E). These findings indicate that ELS caused by MD affects significantly medial entorhinal cortical interneuron dendritic growth later in the adult brain. Subsequently, Sholl analyses were employed to ascertain in which dendritic compartment dendritic complexity is diminished in the maternally deprived interneurons in comparison to the control. The Sholl analysis revealed a significant reduction in dendritic complexity in the proximal dendritic intersections of GAD67-positive interneurons from the maternally deprived animals in comparison to the control group (2-way repeated measures ANOVA, *** *p* < 0.001. Figure 2F). Moreover, the total number of the dendritic intersections of interneuron dendrites was significantly reduced in the maternally deprived interneurons (Figure 2G). These data suggest that early stress caused by MD results in growth deficits later in the adult mEC.

### 2.2. MD Results in a Reduction in the Complexity of Proximal Dendrites of Interneurons in the SSC

**Figure 3:**
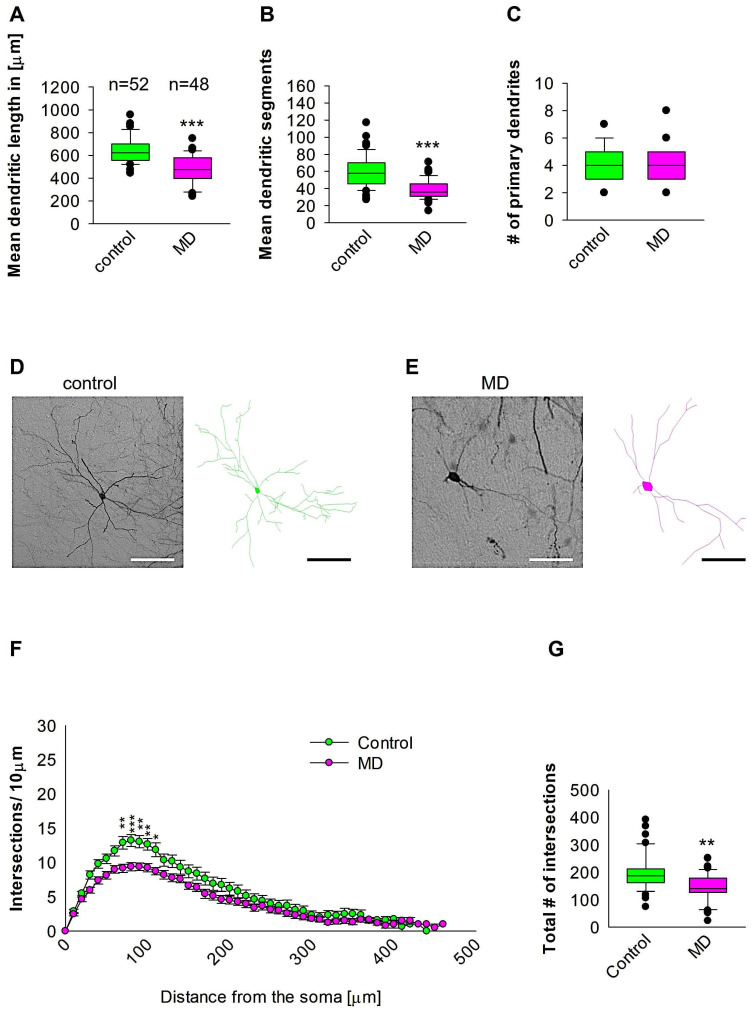
MD effect on the morphology of the interneuron of the SSC. Parasagittal brain slices from the SSC region were stained with EGFP to visualise morphology by light microscopy. GAD67-positive interneurons were reconstructed using Neurolucida. (**A**–**C**,**G**) The median value is represented by the horizontal lines within the box plots, while the variabilities outside the upper and lower quartiles are indicated with whiskers. The middle half of the sample is represented with the box. (**A**) Mean dendritic length. (**B**) Mean dendritic segments. (**C**) Mean of the number of primary dendrites. The number of reconstructed cells is shown in (**A**) for the control and MD groups. The number of cells analysed were obtained from 8 control mice (2–3 slices corresponding only to the SSC area) and 8 MD mice (2–3 slices). Example images at 40× magnification from a control (**D**) and an MD interneuron are shown (**E**). The traces are shown next to the images. Scale bars = 50 μm. (**F**) Sholl analysis of the control and MD groups. The error bars in (**F**) represent the standard error mean. MD-induced decrease in the number of intersections was observed between 70 and 110 μm from the soma using *t*-test post hoc Bonferroni corrections. * *p* < 0.05; ** *p* < 0.01; *** *p* < 0.001. (**G**) The mean of the total number of dendritic intersections. In the statistical analyses conducted for the experiment in (**F**), the number of dendritic intersections was compared between the control and MD groups at each distance point. For the graphs in (**A**–**C**,**G**), the *p* values were calculated using the Mann–Whitney U test and reported only if they were statistically significant. ** *p* < 0.01; *** *p* < 0.001.

We further reconstructed dendritic arbores on GAD57-positive interneurons from the SSC area. Quantitative morphological analyses revealed that MD at the early developmental stage at P9 resulted in a reduction in dendritic length of GAD67-positive interneurons at P60 when compared to the control group (Figure 3A,D,E). In a similar manner, the mean dendritic segments of GAD67-positive interneurons from the maternally deprived mice were also significantly decreased when compared to the control group (Figure 3B,D,E). The number of primary dendrites was unaltered between the two groups (Figure 3C–E). These findings indicate that ELS caused by MD significantly affects the dendrite growth of interneurons in the SSC later in the adult brain. Subsequently, Sholl analysis demonstrated a significant reduction in dendritic complexity solely in proximal dendritic intersections of the GAD67-positive interneurons in the maternally deprived animals when compared to the control (2-way repeated measures ANOVA, *** *p* < 0.001, Figure 3F). Furthermore, the total number of dendritic intersections of interneuron dendrites was significantly reduced in the maternally deprived interneurons (Figure 3G). These data suggest that early stress caused by MD can result in a growth deficit in the dendrites later in the adult SSC.

### 2.3. Early MD Did Not Affect Interneuron Morphology Later in Adult MC

**Figure 4:**
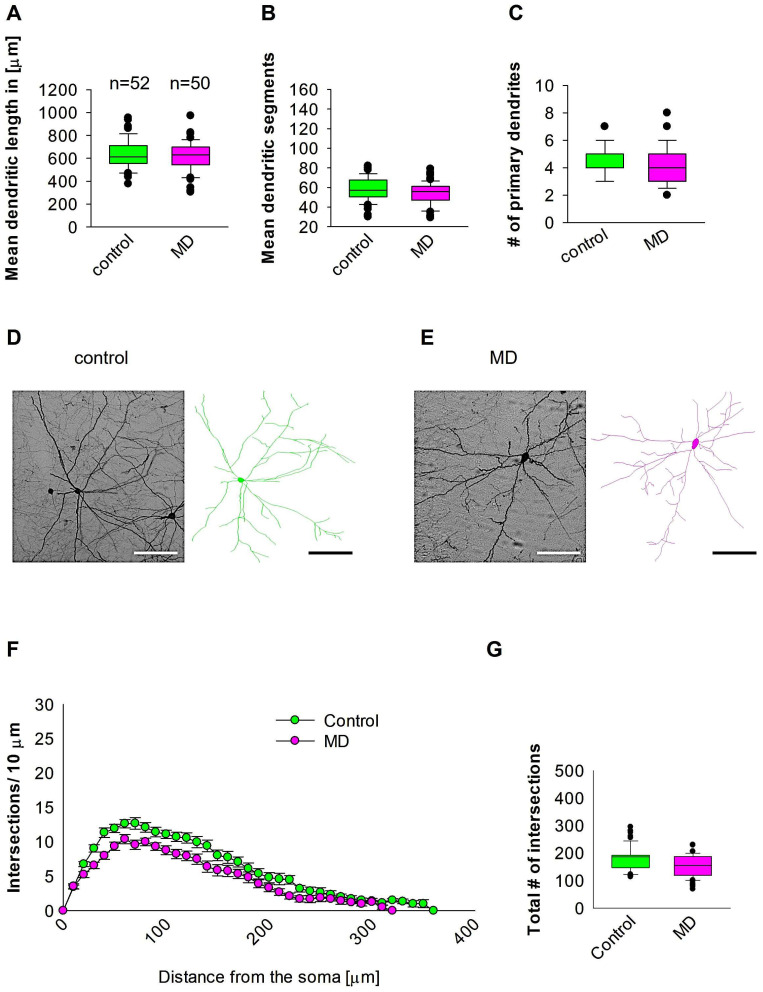
MD does not affect MC interneuron morphology. Parasagittal brain slices from the MC region were stained with EGFP to visualise morphology by light microscopy. GAD67-positive interneurons were reconstructed using Neurolucida. (**A**–**C**,**G**) The median value is represented by the horizontal lines within the box plots, while the variabilities outside the upper and lower quartiles are indicated with whiskers. The middle half of the sample is represented with the box. (**A**) Mean dendritic length. (**B**) Mean dendritic segments. (**C**) Mean of the number of primary dendrites. The number of reconstructed cells is shown in (**A**) for the control and MD groups. The number of cells analysed was obtained from 8 control mice (2–3 slices corresponding only to the MC area) and 8 MD mice (2–3 slices). Example images at 40× magnification from a control (**D**) and an MD interneuron are shown (**E**). The traces are shown next to the images. Scale bars = 50 μm. (**F**) Sholl analysis of the control and MD groups. The error bars in (**F**) represent the standard error mean. MD did not affect the number of intersections using 2-way repeated measures ANOVA. (**G**) The mean of total number of dendritic intersections. In the statistical analyses conducted for the experiment in (**F**), the number of dendritic intersections was compared between the control and MD groups at each distance point.

To investigate whether early MD influence the morphology of interneurons in adult MC, we ran quantitative morphological quantifications. Our analyses showed that MD at the early developmental stage of P9 did not affect the dendritic length of GAD67-positive interneurons in the MC at P60 (Figure 4A,D,E). In a similar fashion, the mean dendritic segments of GAD67-positive interneurons from the maternally deprived mice were also non-significant from the control group (Figure 4B,D,E). In addition, the number of primary dendrites was unaltered between the two groups (Figure 4C–E). These findings indicate that ELS caused by MD does not influence MC interneuron dendritic morphology later in the adult stage. However, Sholl analyses demonstrated no significant change in dendritic complexity of the GAD67-positive interneurons in the maternally deprived animals in comparison to the control group (2-way repeated measures ANOVA, *p* = 0.35. Figure 4F). However, the total number of dendritic intersections of interneuron dendrites was reduced in the maternally deprived interneurons (Figure 4G). These data suggest that early stress caused by MD does not affect interneuron morphology in the MC.

### 2.4. MD Affects Dendritic Growth Complexity of Interneurons in the Hp

**Figure 5:**
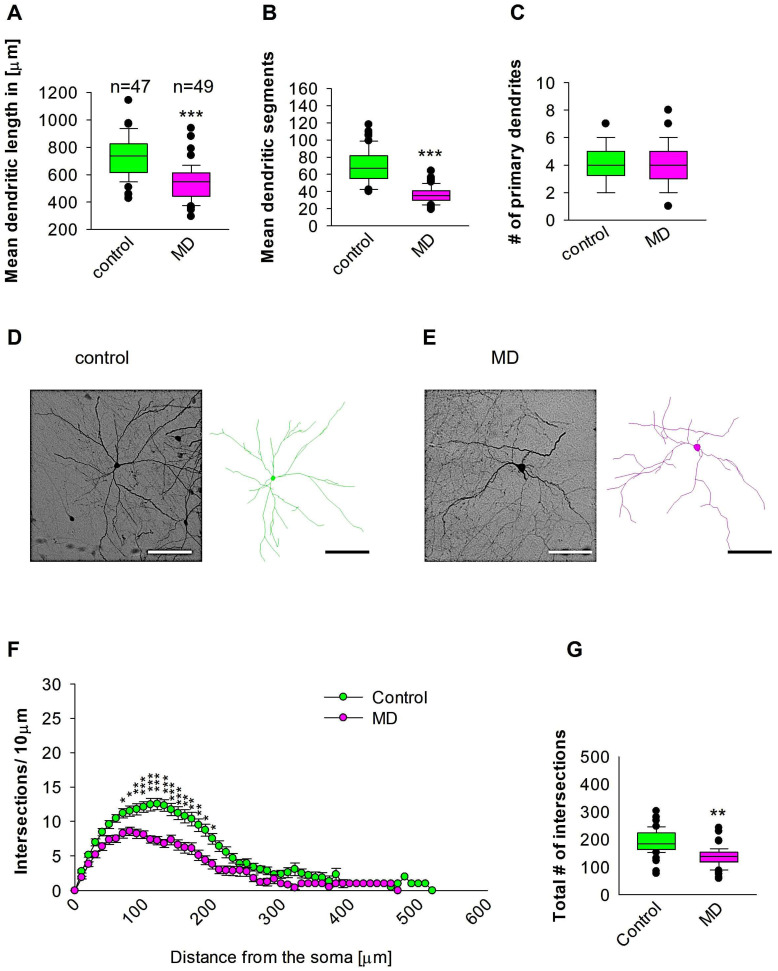
MD effect on the morphology of the interneuron of the Hp. Parasagittal brain slices from the Hp region were stained with EGFP to visualise morphology by light microscopy. GAD67-positive interneurons were reconstructed using Neurolucida. (**A**–**C**,**G**) The median value is represented by the horizontal lines within the box plots, while the variabilities outside the upper and lower quartiles are indicated with whiskers. The middle half of the sample is represented with the box. (**A**) Mean dendritic length. (**B**) Mean dendritic segments. (**C**) Mean of the number of primary dendrites. The number of reconstructed cells is shown in (**A**) for the control and MD groups. The number of cells analysed was obtained from 8 control mice (2–3 slices corresponding only to the Hp area) and 8 MD mice (2–3 slices). Example images at 40× magnification from a control (**D**) and an MD interneuron are shown (**E**). The traces are shown next to the images. Scale bars = 50 μm. (**F**) Sholl analysis of the control and MD groups. The error bars in (**F**) represent the standard error mean. MD-induced decrease in the number of intersections was observed between 70 and 200 μm from the soma using *t*-test post hoc Bonferroni corrections. * *p* < 0.05, ** *p* < 0.01, and *** *p* < 0.001 (**G**) The mean of the total number of dendritic intersections. In the statistical analyses conducted for the experiment in (**F**), the number of dendritic intersections was compared between the control and MD groups at each distance point. For the graphs in (**A**–**C**,**G**), the *p* values were calculated using the Mann–Whitney U test and reported only if they were statistically significant. ** *p* < 0.01; *** *p* < 0.001.

Quantitative morphological analyses in the Hp revealed that MD at the early developmental stage at P9 resulted in a reduction in dendritic length of GAD67-positive interneurons at P60 when compared to the control group (Figure 5A,D,E). In a similar manner, the mean dendritic segments of GAD67-positive interneurons from the maternally deprived mice were also significantly decreased when compared to the control group (Figure 5B,D,E). However, the number of primary dendrites was unaltered between the two groups (Figure 5C–E). These findings indicate that ELS caused by MD significantly affects the dendrite growth of interneurons in the Hp region later in the adult brain. Further Sholl analysis revealed a significant reduction in dendritic complexity exclusively in the proximal dendritic intersections of the GAD67-positive interneurons of the Hp region in the maternally deprived animals when compared to the control (2-way repeated measures ANOVA, *** *p* < 0.001. Figure 5F). In addition, the total number of dendritic intersections of interneuron dendrites was significantly reduced in the maternally deprived interneurons (Figure 5G). These data indicate that early stress caused by MD can result in a dendritic growth deficit in the interneurons of the adult Hp.

### 2.5. Early MD Reduces GAD67-Positive Interneurons in Adult mEC and Hp

**Figure 6:**
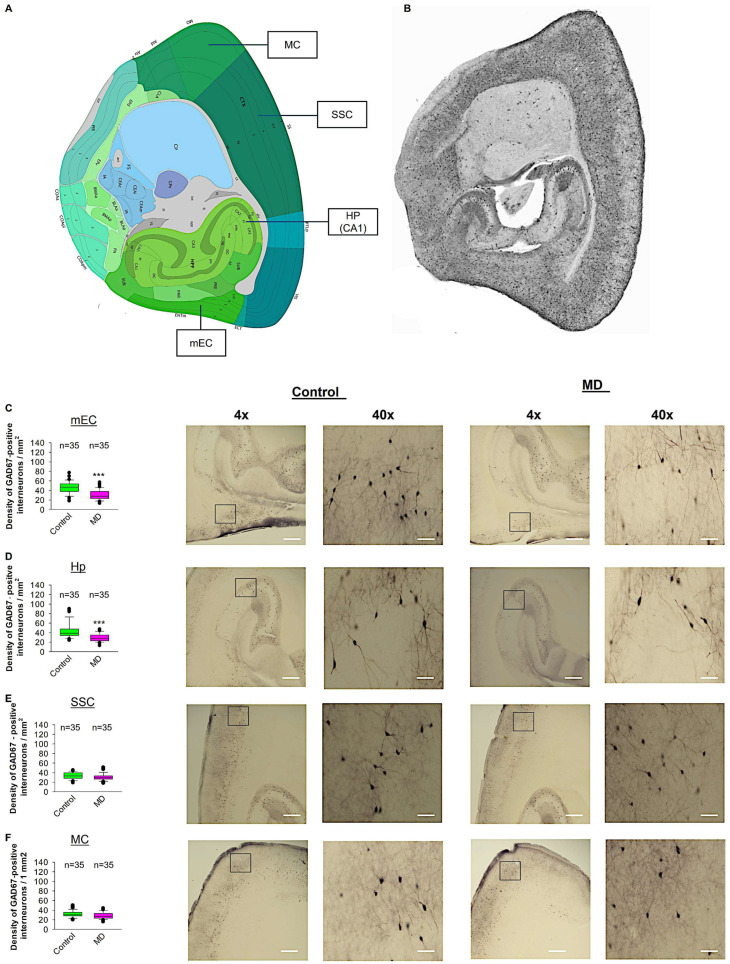
Effect of MD on GAD67-positive interneuron density. (**A**) Virtual parasagittal brain slice from the Allen Brain Atlas reference showing the 4 main regions examined. (**B**) An example of a parasagittal brain slice from a GAD67-GFP-stained brain slice. (**C**–**F**) The median value is represented by the horizontal lines within the box plots, while the variabilities outside the upper and lower quartiles are indicated with whiskers. The middle half of the sample is represented with the box. (**C**) Mean values of the number of GAD67-positive interneurons per 1 mm^2^ in the mEC region. The accompanying illustration depicts a photomicrograph captured at 4× magnification (scale bar = 200 μm) and a zoomed area captured at 40× magnification (scale bar = 20 μm) from the control and MD mice from the mEC region. It is presented alongside the corresponding graph. (**D**) The box plot in the graph represents the mean values of the number of GAD67-positive interneurons per 1 mm^2^ in the SSC region. (**E**) Mean values of the number of GAD67-positive interneurons per 1 mm^2^ in the MC region. (**F**) Mean values of the number of GAD67-positive interneurons per 1 mm^2^ in the Hp region. The total number of the analysed regions of interest (ROIs) is indicated above the box plot for each condition. 5–6 ROI were analysed per animal from control and MD groups. The number of analysed ROIs is shown in the graphs for the control and MD groups. The analysed ROIs were obtained from 7 control mice (2–3 slices corresponding to each area) and 8 MD mice (2–3 slices corresponding to each area). Mann–Whitney U test, *** *p* < 0.001.

To test whether ELS caused by MD influences the density of GAD67-positive interneurons in the neocortex, we performed profile densities (number of GAD67-positive interneuron cells per surface area) in parasagittal slices from both brain hemispheres. We counted GAD67-positive interneurons from the mEC, SSC, MC, and Hp (Figure 6). In the mEC and Hp, the profile density of GAD67-positive interneurons in the maternally deprived brain slices was significantly reduced compared to the control group (Figure 6C,D). In the SSC and MC, the profile density of GAD67-positive interneurons in the maternally deprived brain slices did not differ from the control group (Figure 6E,F). We conclude that MD reduces the number of GAD67-positive interneurons in the mEC and Hp, but not in the SSC or MC.

## 3. Discussion

The current study examined the long-term effects of MD on the dendritic morphology of GABAergic interneurons in the mEC, SSC, MC, and Hp. The results demonstrated a significant reduction in dendritic length and branching in the maternally deprived male mice in the mEC, SSC, and Hp, but not in the MC. This finding is novel, as the long-term impact of ELS induced by MD on the dendritic growth of inhibitory interneurons has not been previously investigated. The P9 MD treatment has been shown to be an effective protocol for inducing pronounced effects in adult mice. A single human year is often considered equivalent to nine mouse days [41], though the comparison is not straightforward due to the differing life cycles of mice and humans. It is important to note that in both species, an age of nine days in a mouse and one year in a human corresponds to a significant developmental milestone in somatosensory and motor activity.

Dendrites, the main input compartment of the central nervous system, develop under the influence of both intrinsic and extrinsic factors [42]. Over the past three decades, numerous studies have highlighted how dendritic growth is responsive to external influences, which can affect both the local and global mechanisms of dendrite development [43]. ELS has been shown to affect dendritic morphology within the brain. For example, ELS induces spine loss and dendritic atrophy in the PFC, and these changes can be reversed by a high-fat diet [44]. Additionally, MD has been linked to a reduction in the expression of BDNF and immediate early genes in the hippocampus [45], resulting in a reduction in mature spine density in the apical dendrites of CA1 pyramidal neurons. Interestingly, in our study, MD did not impact dendritic morphology in the MC, which contrasts with the findings from other areas of the brain, suggesting that the effects of MD on dendritic growth might be region-specific. In contrast, repeated MD between postnatal days 6 and 21 (3 h daily) has been found to reduce dendritic arborization in pyramidal neurons and to induce anxiety-like behaviours [46]. Conversely, other studies have demonstrated an increase in dendritic growth and spine density in subregions of the PFC following MD [47], further supporting the notion that the effects of MD on dendritic growth may vary by brain region and neuron type. Our data align with this view, showing region-specific changes in dendritic morphology in the mEC and Hp, suggesting that these regions may be particularly vulnerable to the effects of early-life adversity.

Defects in the growth of neocortical interneurons can lead to pathological hyperexcitability in neuronal circuits, which may contribute to the development of disorders such as epilepsy, schizophrenia, and bipolar disorder [38]. Reelin, an important extracellular matrix protein, plays a critical role in neuronal migration and cortical development. Reelin deficiency has been associated with altered interneuron growth, which could affect neuronal circuit functionality [48,49,50]. Moreover, the number of reelin-expressing interneurons was found to be reduced in the hippocampal CA1 and CA3 regions of MD rats [8]. Reelin has been shown to regulate the dendritic growth of interneurons in both the somatosensory cortex and mEC [49,50], which supports the idea that MD-induced ELS can impact interneuron development, leading to potential long-term neurological impairments.

Our study also revealed that MD reduced the number of GAD67-positive interneurons in the adult mEC and hippocampus. While the expression of GAD67 itself was not significantly affected by MD, our findings are consistent with previous studies showing a reduction in the density of specific interneuron markers following MD, including Parv and calbindin [8]. This reduction in interneuron density may contribute to altered circuit activity, potentially leading to the behavioural changes associated with stress and anxiety. In line with these findings, previous research has reported that alterations in the PNN surrounding Parv-positive interneurons are a consequence of early stress [51]. Interestingly, some studies have also suggested that MD induces region- and sex-specific effects on the maturation of PNNs, particularly in the ventral hippocampus [52]. Additionally, the repeated MD with the early weaning model has been shown to induce anxiety-like behaviour and altered activity levels, as well as changes in PNN intensity around Parv-positive interneurons in the ventral hippocampus [53]. While there is evidence that MD may cause sex- and age-specific changes in behaviour and neural parameters [54], we observed that in our model, MD resulted in a significant reduction in the number of interneurons in the mEC and hippocampus, along with increased oxidative stress [55]. The combination of these factors may contribute to a reduction in the survival of interneurons in adulthood, highlighting the vulnerability of these cells to early-life adversity during key developmental windows. The importance of this research lies in its ability to identify the region-specific effects of MD on neuronal morphology and its potential to contribute to a broader understanding of the neurological consequences of early-life adversity. By focusing on the role of GABAergic interneurons and the impact of MD on their dendritic growth and density, this study lays the groundwork for future research exploring therapeutic interventions aimed at mitigating the effects of ELS on brain development and function. Understanding these mechanisms is crucial for developing strategies to prevent or treat neurodevelopmental and psychiatric disorders linked to early-life stress.

## 4. Material and Methods

### 4.1. Ethics Statement

All the experiments conducted in this study were reviewed and approved by the local ethics committee. The requisite licence for animal experimentation was obtained from the United Arab Emirates University Animal Ethics Committee of the United Arab Emirates University under the permission number ERA_2023_3852.

### 4.2. Animals and MD Procedure

The animals were maintained under a standard 12 h light cycle and were provided with food ad libitum in accordance with standard mouse chow specifications. A male and three nulliparous 6-month-old GAD67-EGFP mice [56] were placed together in a standard plexiglass cage in a temperature-controlled room. The subjects were provided with unlimited access to water and food. Two weeks later, the male was removed, and the dams were checked daily for delivery as they became pregnant. The day of delivery was designated as postnatal day zero (P0). On P9, the litters were weighed and then subjected to the MD procedure, as previously published [13,57]. The dams were removed from the litter, and the pups were maintained in their home cage at room temperature. Following a 24 h interval, the pups were weighed once more, and the dams were returned to their cages. A control experiment was conducted in which the dams were removed from their home cages for a period of five minutes. The litters were allowed to develop until postnatal day 21, at which point they were weaned and allocated to new cages according to their sex. To prevent the occurrence of sexual dimorphism, only male GAD67-EGFP mice were utilised for morphological studies [58], a practice that has been employed in numerous previous studies [59,60]. The animals were sacrificed at P60, a stage that corresponds to young adulthood. This study was not pre-registered. No exclusion criteria were pre-determined and the study was exploratory. Randomization was performed to allocate the subjects in the study. No sample size calculation was performed in this study.

### 4.3. Immunohistochemistry

The animals were deeply anaesthetised with 5% isoflurane and perfused transcardially with aerated phosphate-buffered saline (PBS) followed by a fixative containing 4% paraformaldehyde in 0.1 M phosphate buffer (pH 7.2). The MD and control brains were removed immediately after perfusion and postfixed overnight in the same fixative. A vibratome was then employed to obtain 150 μm parasagittal sections from both hemispheres of the lateral entorhinal cortex to the midline. The Allen Brain Atlas (https://mouse.brain-map.org/static/atlas (accessed on 19 November 2024)) was utilised as a reference to collect brain slices from the mEC, Hp, SSC, and MC. The entire left and right cerebral hemispheres were serially sectioned from the lateral side to the midline. A total of 16 sections were selected from the serial section wells of each hemisphere of each animal, ensuring that these sections were taken from the same serial section of all the animals (8 control and 8 MD mice). For Figure 6, the 16 slices were divided into four regions (mEC, SSC, MC, and Hp). For the SSC, we used the slice approximately from 0 to +2 mm; for the hp from −1 to −3 mm; for mEC -from −4 to −5 mm; and for MC from +1 to +4 mm in relation to bregma. Five to six slices from the cortical region were analysed. In each cortical region, three different areas of interest were selected, and the mean was calculated. This mean value was plotted on the graph. For Figure 2, Figure 3, Figure 4 and Figure 5, four to six cells were selected from each region per animal. Subsequently, immunohistochemistry was conducted using the anti-EGFP antibody to convert EGFP into a stable visible 3,3′-diaminobenzidine (DAB) staining, thereby enhancing resolution for 3-D reconstruction. Initially, an elimination of endogenous peroxidase activity was performed with 0.3% hydrogen peroxide in PBS at room temperature for 1 h. Following several washes with TBS (Tris-buffered saline: 50 mM Tris, 150 mM NaCl, pH 7.6), and permeabilization in TBST (TBS, 0.1% Triton X), the brain slices were blocked for one hour with 1% normal goat serum in TBST. The brain slices were incubated for 24 h at room temperature with the primary antibody, chicken anti-GFP (1:8000, Abcam, Cambridge, United Kingdom, ab13970, RRID:AB_300798). Following two washes in TBS, the donkey anti-chicken biotinylated antibody (1:300, Cat# 703-065-155, Jackson ImmunoResearch; West Grove, PA, USA, RRID:AB_2313596) was applied for three hours at room temperature. Following this, the slices were washed several times in TBS buffer and the extravidin–peroxidase conjugate was added for another hour. To visualise any labelled neurons, the slices were incubated for 10 min in a solution of 25 mg diaminobenzidine (DAB) in 50 mL 0.1 M phosphate buffer (PB, pH 7.4) with 7.5 μL hydrogen peroxide (30%).

### 4.4. Quantitative Analyses of GAD67-Positive Interneurons

Parasagittal brain serial sections (150 μm) were used to define the borders of the mEC, SSC, MC, and Hp regions. The profile density of GAD67-positive interneurons was estimated by counting the EGFP-immunolabeled neurons within the delineated region of interest (ROI). Images of the cell body area were captured on a light microscope (Zeiss, Oberkochen, Germany) with a 40× objective using a 1 mm^2^ grid. The counting of GAD67-positive neurons was performed manually using a 1 mm^2^ grid. Only those cells that exhibited well-defined staining and a discernible nucleus were included in the counting. It is also noteworthy that the analysts were blinded to the conditions, which ensured that no bias was introduced throughout the process.

### 4.5. Three-DimensionalNeuron Reconstruction of Interneurons

EGFP-immunostained interneurons were reconstructed with the Neurolucida system (MicroBrightField, Williston, VT, USA) at 1000× magnification. To quantify GAD67-positive interneuron morphology, a series of parameters were calculated. These included mean dendritic length (total dendritic length divided by the number of primary dendrites), mean number of dendritic segments (total number of dendritic branches divided by the number of primary dendrites), and number of primary dendrites. For the three-dimensional reconstruction of brain regions, the following areas were considered: mEC, SSC, MC, and Hp. The Sholl analysis was conducted to identify the area where dendritic complexity changed by examining the number of dendrite intersections at 10 µm interval distance points starting from the cell soma.

### 4.6. Statistical Analysis

The statistical analyses were conducted using Sigma Stat 12 (SPSS Incorporated). Comparisons between the two groups were conducted using either Student’s *t*-test or the Mann–Whitney test, depending on whether the equal variance test passed. The results were deemed statistically significant at the *p* < 0.05 level. For the Sholl dendritic intersection analyses, we performed a 2-way repeated measures ANOVA with treatment (MD vs. control) as the between-group factor and radial distance from the soma. Violation of the sphericity assumption for repeated measures was corrected using the Greenhouse–Geisser correction for degrees of freedom [61]. At each distance interval between the control and MD groups, *t*-tests post hoc with Bonferroni correction were performed.

## 5. Conclusions

This study underscores the long-term consequences of early-life stress induced by MD on the development of GABAergic interneurons, specifically in the mEC, SSC, and Hp. Our findings demonstrate that MD leads to a reduction in dendritic length and branching of interneurons, and a significant decrease in the number of GAD67-positive interneurons in these regions. These changes highlight the profound impact of ELS on brain structure and function, with potential implications for the development of psychiatric and neurological disorders, such as anxiety, depression, and cognitive deficits, in adulthood.

## Data Availability

Data is contained within the article.
